# Facilitating engagement: Individual meaning-making and relationship-based trust in women’s experiences of cervical screening

**DOI:** 10.1177/13591053251400069

**Published:** 2025-12-26

**Authors:** Rose Rickford, Melanie Rogers, Rebecca Gunn, Lucy Wearmouth, Abigail Halliday, Susanna Kola-Palmer

**Affiliations:** 1 University of Surrey, Guildford, UK; 2 University of Huddersfield, UK; 3 Kirklees Council, Huddersfield, England, UK; 4 Arden University Coventry, UK

**Keywords:** cervical cancer screening, human papilloma virus, decision-making, person-centred care, health beliefs

## Abstract

Cervical screening rates in the UK are declining. While previous research has identified barriers to screening uptake, less is known about why many women attend cervical screening despite these barriers. This qualitative study explores factors influencing cervical screening decision-making. Through reflexive thematic analysis of semi-structured interviews (*n* = 44), two overall themes were developed: Perceived purpose of Screening and Experience of Screening. The analysis indicates that beliefs are central to decision-making and relate to individual meaning-making in relation to knowledge and information. The same information may be interpreted differently by different women and therefore impact cervical screening decision-making in different ways. Women associate cervical screening with pain, embarrassment and potential violation, but many choose to attend despite this. Trust, person-centred care and continuity of care are important for mitigating these negative perceptions and experiences. Implications of findings are discussed and include moving beyond knowledge-based public health interventions for enhancing cervical screening uptake.

## Introduction

Cervical cancer causes over 300,000 deaths each year and is the fourth most common cancer among women globally (Cohen et al., 2019; World Cancer Research Fund, 2024). Cervical cancer incidence has decreased by more than 50% in the past 50 years due to screening programmes, and recent reductions in mortality among young women are largely attributed to vaccination against Human Papilloma Virus (HPV; Siegel et al., 2024). As cervical cancer is largely preventable, increasing coverage and uptake of screening and HPV vaccination is imperative for preventing incidences and deaths.

In the United Kingdom (UK), cervical screening is offered to women aged 25–64 years, with screening intervals determined by age and country of residence. Screening currently prevents approximately 70% of cervical cancer deaths in England, with potential to reach 83% if full participation were achieved (National Institute for Health and Care Excellence, 2022). These figures underscore the importance of achieving high participation rates.

Cervical cancer screening coverage has been decreasing in England for a number of years – in 2024–25 coverage was 68.8%, compared to 80.3% in 2004–2005 (Department of Health and Social Care, 2025). There is evidence of socio-demographic inequalities in screening attendance, with minoritised ethnic women (Marlow et al., 2015), lesbian women (Saunders et al., 2021), women with learning disabilities (Reynolds et al., 2008) and women living in areas of high deprivation (Urwin et al., 2024) less likely to attend. A longitudinal study looking at cervical screening attendance in England from 2013 to 2022 found that practices with larger patient lists tend to have lower cervical screening attendance compared to those with smaller patient lists, and practices with high ratios of practice nurses to patients tend to have higher cervical screening attendance compared to those with low ratios of practice nurses to patients (Urwin et al., 2024). There is evidence that difficulty securing convenient appointments is also significantly associated with screening non-attendance (Marlow et al., 2015; Waller et al., 2009).

In addition to these structural and service level factors, individual beliefs and feelings about cervical cancer, HPV and cervical screening can be barriers to attendance. These include perception of low personal risk (Chorley et al., 2017; Marlow et al., 2019; Waller et al., 2009), fear of a cancer diagnosis (Marlow et al., 2015, 2019), belief that cervical cancer is caused by sexual promiscuity (Chorley et al., 2017), and belief that stigma is attached to cervical cancer (Vrinten et al., 2019). The experience of screening itself has been found to be important for women with reports of strong emotional and physical experiences during cervical screening, including pain, fear, shame and vulnerability (Chorley et al., 2017). Several studies have found that negative past experiences, pain and embarrassment are reported barriers to attending screening (Gillibrand et al., 2025; Marlow et al., 2019; Taratula-Lyons and Hill, 2024; Waller et al., 2009; Wilding et al., 2020), but there is also evidence that these are not actually predictors of non-attendance (Waller et al., 2009; Wilding et al., 2020), highlighting a paradox between perceived and actual behavioural influences. There are still gaps in understanding why some women attend despite barriers, while some do not. This study addresses this gap by exploring how women make sense of such perceptions in the context of their screening decisions.

This paper presents a reflexive thematic analysis of factors that women draw upon when explaining their decisions about cervical screening attendance. While previous research has identified a range of potential barriers to screening attendance, little is known about why some women choose to attend when others opt out despite similar barriers. A more nuanced understanding of the personal, relational and systemic factors which facilitate screening engagement is relevant for informing public health interventions.

## Methods

### Study design

The qualitative analysis presented here is drawn from a wider mixed-methods study about women’s attitudes to cervical screening, including attitudes to proposed changes in screening frequency. Findings relating to attitudes to proposed changes in screening frequency are reported elsewhere (Kola-Palmer et al., 2022; Rickford et al., 2023). This paper presents a reflexive thematic analysis based on semi-structured one-on-one interviews, addressing the question *What matters to women in in the UK when deciding whether to attend cervical screening?*

### Sampling and recruitment

Participants were selected using random sampling from a pool of respondents to a cervical screening and HPV survey who had agreed to be contacted for follow-up. Inclusion criteria for the online survey were being aged 18–45 years and living in the UK. Women below the age of screening eligibility were included because they will be affected by reduced screening frequency, and their inclusion allowed us to obtain a broader range of perspectives relevant to future engagement with the screening programme. Of the 801 women who accessed the survey, 90 (11.2%) participants consented to be contacted for the follow-up interview. Fifty-one women were sent full study information with invitations to participate, and data saturation was confirmed by the team after 44 interviews.

### Data collection

Interviews were held in 2019. Ethical approval was granted by the host institution’s ethical review board (approval number SREP/2018/047). All participants provided written and oral consent. To protect participants’ privacy, confidentiality is maintained through data protection procedures, and quotations are presented using pseudonyms.

Interview procedures have been described elsewhere (Rickford et al., 2023). Participants were interviewed face-to-face or by phone by one of three researchers (MR, AH, SKP), with interviews digitally recorded. The interview schedule was based on illness perceptions and health beliefs and included questions relating to awareness, attitudes and beliefs about HPV vaccination, cervical screening and cancer. Interviews lasted on average 51 minutes.

### Participants

The dataset includes 44 semi-structured interviews with UK-based women aged 18–45 years (Table 1). Of these, 82% (*n* = 36) were eligible for cervical screening and had received an invitation, while 18% (*n* = 8) had not yet been invited, as they were below the minimum age of screening in the UK (25 years). All eligible participants had attended screening at least once (*n* = 36). Among those screened, 39% (*n* = 17) had received an abnormal result or a positive HPV test, and 5% (*n* = 2) had been diagnosed with cervical cancer diagnosis (Table 1).

**Table 1.** Frequencies of age categories, screening history and experience.

Variable	*N*	Yes (*n*)	No (*n*)	N/A (*n*)
*Age category*				
18–24	121			
25–45	32			
*Screening history and experience*				
HPV vaccinated		16	28	0
Previous screening		36	8	0
Attends per recommendations		35	1	8
Always attended per recommendation		31	5	8
Abnormal screening result and/or positive HPV test in lifetime		17	19	8
Cervical cancer diagnosis in lifetime		2	42	0

*Note*. Four under-25s had received screening invitations, due to clinical vulnerability (*n* = 3) and screened just prior to reaching screening age, with interview close to 25th birthday (*n* = 1).

Regarding HPV vaccination, 17 participants had been offered the vaccine, with 16 accepting and one declining (Table 1).

### Analytic method

Reflexive thematic analysis (RTA; Braun and Clarke, 2021), was conducted by the first author. The analytic process involved coding and recoding the data to observe patterns and consider different possible interpretations. The codes were grouped into themes in relation to the research question: “*What matters to women when deciding whether or not to attend cervical screening?*” The whole dataset was recoded into draft themes to check their power to represent the content of the data. The themes were then adjusted, data recoded, and themes adjusted again, in an iterative process until the final analysis was produced.

The aim of RTA is not to provide a complete description of the dataset, or to present the only objectively “correct” analysis. Instead, we draw on Maxwell’s (1992) typology of qualitative validity. We aimed for descriptive validity (a description of participants’ perspectives that a reasonable reader, on examining the same data, would agree was valid); and interpretative validity (an account of participants’ perspectives that we could reasonably expect that the participants themselves would agree with, based on the content of the data). These forms of validity have been tested through the iterative process of checking and rechecking codes, grouping and regrouping codes into themes and constantly reflecting on the ability of the analysis to produce a valid account of the dataset in relation to the research question.

### Reflexivity statement

The study was designed by SKP and MR because of a conviction that women’s voices matter in women’s healthcare. We are an interdisciplinary team with diverse experience relating to cervical screening, including as psychologists/sociologists (RR, AH, SKP), an Advanced Nurse Practitioner and nursing scholar (MR), public health professionals (RG, LW) and patients (all authors). We drew on personal and professional experience during the semi-structured interview process, which means that interview data were shaped by the various positionalities of the three interviewers – as a sample taker (MR), an experienced feminist health psychologist (SKP) and a young woman who had not yet attended cervical screening (AH). Similarly, the RTA was informed by RR’s feminist perspective, while having a diverse team with whom to discuss the developing analysis helped to enrich it by drawing in a variety of personal and professional viewpoints.

## Findings

There are two overall themes describing the broad factors that women consider when making decisions about cervical screening attendance. These are *Perceived Purpose of Cervical Screening* and *Experience of Cervical Screening*. In relation to perceived purpose of screening a perceived benefit was that screening was understood as potentially lifesaving. This was the most cited reason for attending screening. Barriers that related to the perceived purpose of screening included assumptions that screening was not necessary, fear of a cancer diagnosis, and a belief that attendance at screening was stigmatising. A mitigating factor was understood to be knowledge of the purpose of screening.

Experience of screening was commonly perceived as a barrier to attendance. Some women described negative experiences of attending screening, or, in the case of women who were yet to attend, anticipated negative experiences. While all but one currently attended as advised, negative experiences were perceived as the most likely reason that other women do not attend. For the one participant who did not currently attend as advised, the reason given was the negative experience of the screening procedure. Negative experiences included pain, embarrassment/shame and vulnerability/violation, which were presented by participants as potential reasons for non-attendance at cervical screening. Participants also suggested ways in which these negative aspects of the experience can be mitigated and thereby made less significant in the process of deciding whether to attend cervical screening. These mitigating factors included the speed of the test, person-centred care, continuity of care and knowledge of the procedure.

### Theme 1: Perceived purpose

#### Benefit: Screening as lifesaving

The main reason given for attending screening was understandings of its purpose as potentially lifesaving. This was mentioned by women who attend as advised, women who have not yet been invited to attend, and the one participant who does not attend as advised. Participants perceived that cervical screening is “important” (Victoria, Helen, Harriet Pat, Sophie, Angela, Elspeth), “lifesaving” (Tessa), that it could “save your life” (Queenie, Denise, Tina, Sharon) and that not attending could be “killing yourself” (Helen). Some said that being screened is “reassuring” (Dana) and provides “peace of mind” (Yvonne). Screening was perceived as something that can “protect” (Angela) or provide “prevention” (Queenie) from “preventable” (Becky) cervical cancer, and as offering an opportunity to “catch it earlier” (Jane), “catch it at a very early stage” (Elspeth) and a “chance to stop it” (Helen). As Anita put it, “the earlier you find things out, the quicker you get treatment, and hopefully the better the results are.” Women who had received the HPV vaccination considered screening to still have protective value for them, based on their understanding of the function of screening combined with their understanding of the function and efficacy of the vaccine. Yvonne attends screening because “nothing is 100%,” and Jean felt “it’s better to be safe than sorry.” Maeve said she sees the vaccine as providing protection from HPV, but screening as a way to identify cancer, and that “I don’t think HPV is the only way you can get cancer.” As reported elsewhere (Rickford et al., 2023), there seems to be misunderstandings around the preventive versus diagnostic function of screening. Some women in this study understood cervical screening as a test for cancer, while others understood it as a preventative measure. Irrespective of this difference in understanding, most participants saw screening as an important way to protect their health.

#### Barrier: Belief of low personal risk

As all but one of the participants attended screening as recommended at the time of the interviews, or were planning to do so when invited, most were not able to comment from a current first-person perspective about reasons women do not attend screening. However, some spoke about reasons they had not attended in the past, and most spoke about their perceived reasons that other women do not attend. While most of the reasons given related to experience of screening (Theme 2), participants reported that a lack of concern about cervical cancer may also be a reason for non-attendance. Vera believed that some women miss screenings because they “think [cancer] is not going to happen to them.” Two participants reported that a perception of themselves as low risk was the reason for their non-attendance earlier in their lives. Renee said she missed screenings in her20s because she felt “invincible,” and Rebecca, the one participant who does not currently attend as recommended, said that she did not attend when first invited because she had had few sexual partners and therefore had an “I’m sure I’m fine attitude.” (Rebecca’s current reason for non-attendance relates to the experience of painful screening, following treatment for cervical cancer. This is discussed under Theme 2).

#### Barrier: Fear of diagnosis

Some women suggested that understanding screening as a test for disease, combined with personal deep fear of diagnosis of disease, might explain non-attendance in some cases. Elspeth and Vera both spoke about women in their lives whose fear of receiving a cervical cancer or HPV diagnosis meant that they avoided screening.

“Her perspective is fear because she knows she doesn’t look after herself. She knows she should have done. She knows she should have done and it is fear, so she puts her head in the sand” (Elspeth, speaking about her mother)

“They are just afraid of it, and they are even afraid of taking the test to know if they have it or not” (Vera, speaking about women she works with)

#### Barrier: Stigma

Vera said that some women she knows believe that “only certain group of people will take it [screening] because they are doing things that are immoral.” She explained that in her country of origin (Iran) there is stigma associated with seeking screening if you are not married. She reported that, in Iran, “finding a doctor that respects you and . . . doesn’t judge you, is not easy,” and some women carry this sense of stigma with them when the move to the UK. Vera and Gillian (both suggested that some women believe that cervical screening results affects “virginity” (Vera), which can be a barrier to attendance.

Rebecca, a woman who has been treated for cervical cancer and who has missed a number of screenings (before and after the diagnosis), explained that she had felt “a great deal of shame attached to the disease.” This was echoed by women who had received positive HPV tests who said they felt “dirty” (Angela, Denise) “disgusted” (Angela) and a sense of “stigma” (Harriet), and Yvonne, who had not yet been invited for screening, suggested that women who are diagnosed with HPV “might feel a bit of shame.”

#### Mitigating factor: Understanding of screening purpose

Women perceived that lack of understanding about the purpose of screening was a reason for non-attendance. Some of those who had had periods of non-attendance said that they had not seen screening as “important” (Cindy, Rebecca) or a “priority” (Cindy). This meant they had not perceived the “it can save your life” benefit to be of sufficient relevance to them to persuade them to attend. Rebecca, who had a history of cervical cancer and had not attended screening when first invited, said the purpose of screening “wasn’t really pushed home. . .It is certainly nothing that was ever discussed with me.” Speaking about other women, Yvonne suggested that those who do not attend might think “I don’t understand why I have to go or this” and therefore “put if off because you don’t understand it.” Similarly, some suggested that other women may choose not to attend because of a perception that the HPV vaccine provides complete protection against cervical cancer.

“I think it is because there is no education around the HPV vaccine at all. I think that there is a perception around that once you have had the HPV vaccine, you will not get cervical cancer” (Paula)

Most of those who had received the immunisation felt that there was little information available to them at the time.

“I just got told its the HPV vaccine you’re going for, it decreases your chances. That’s it, that’s all I got told so I just sat down and had it.” (Yvonne)

It was felt that “education” (Elspeth, Paula, Victoria, Maeve) and “public information campaigns” (Renee), particularly in schools and on social media, is needed to counter “how little information there is out there” (Beth) about both the purpose of cervical screening and the efficacy of the HPV vaccine in protecting against cervical cancer. Women felt that this would help others to develop the knowledge to make informed choices about screening attendance.

Women also saw knowledge as a way to reduce to fear and worry about cancer and HPV, and thus as a solution to this barrier to attendance. Vera explained that in her work with Iranian women, providing a trusted source of information helps women to feel less afraid of receiving an HPV diagnosis:

“They have this picture that it is something as dangerous as HIV and you will get it and you will die. You cannot do anything about it…. They have not understood it very well.” (Vera)

Similarly, some women reflected that knowledge is important in reducing the stigma women feel about cervical cancer and cervical screening. Speaking about her positive HPV tests, Angela said that she initially felt it was “disgusting” and “dirty” but that “once I’d calmed down and read all the information. . .I was like ‘It’s OK. It doesn’t matter’”. Yvonne’s perception was that women might feel “shame” about an HPV diagnosis “if they weren’t educated properly.”

Overall, the potentially lifesaving purpose of screening was presented as the most important factor in persuading women to attend, while a sense of invincibility to cancer/irrelevance of screening, and fear and stigma associated with HPV and cervical cancer, were considered to be disincentives to attendance. Women perceived that knowledge and understanding of the purpose of cervical screening, and about cervical cancer and HPV, could encourage attendance by increasing the belief that screening could be lifesaving, while decreasing the significance of stigma. However, some understood cervical screening as a test for a disease which they perceived as stigmatising. This suggests that individual understanding and meaning-making is important in relation to the ability of knowledge to motivate attendance.

### Theme 2: Experience of cervical screening

#### Barrier: Pain and/or physical discomfort

Although some women reported that screening has “never been uncomfortable” (Dana), “doesn’t hurt particularly” (Sharon) or is “painless” (Queenie), most participants mentioned that they find screening painful or physically uncomfortable, or, for those who have not yet attended, that they expect it to be painful or uncomfortable.

For several women, this was balanced against benefits of screening, and the duration of the procedure, in decision-making explanations. Zara and Gillian both described screening as painful and attend as advised, and Fran, who is not yet aged 25, expects it to be painful but plans to attend when invited.

“This moment when they put the brush inside. . . That is really, really painful. . . I didn’t like it. . . it’s really uncomfortable. . .. But it’s just like a few minutes, so if you want to know about your body. . . you can just survive those few minutes.” (Zara)

“I might find it a bit discomforting and a bit painful, but rather that than. . . not” (Gillian)

“I think about the pain of having a smear test, although I will have it done. I’d never not have it done. . .it is just over in a second and it is really worth it. So, although I do think about that, it would never stop me from going to have one”. (Fran)

Three women with a history of gynaecological surgery reported extreme pain. Fiona, who has been treated for cervical cancer, said that smear tests are now “excruciatingly painful” and “unbearable.” Lauren, who has a medical condition that puts her at high risk of cervical cancer, resulting in her requiring six-monthly screenings, said she is “terrified” of the “pain that I am going to experience in the aftermath,” and that she has to take a day off work to recover from cervical screening. Although severe pain affects both Lauren and Fiona’s experience, they choose to attend screening because “it’s going to benefit me” (Lauren) and “you just have to get on with it” (Fiona). However, a third woman, Rebecca, who has been treated for cervical cancer in the past, was the only woman in the study who does not currently attend as recommended. She gave pain among her reasons.

“My cervix doesn’t respond the same way that other people’s do, because it is all scar tissue, so it doesn’t stretch. . .When they are trying to crank you wider, the pain even just that causes is immense.” (Rebecca)

#### Barrier: Embarrassment/shame

While some women reported that “I don’t think it really embarrasses me” (Maureen), “I don’t ever really feel embarrassed” (Helen) and “I am no longer embarrassed” (Fiona), more said that they find, or expect to find, cervical screening “embarrassing” (Angela, Denise, Rosie, Sharon), “awkward” (Indie, Lauren, Dana) and that they get “embarrassed” (Anita, Jean). When asked for their views on the decline in screening attendance, women suggested that “maybe people are a little bit embarrassed” (Becky), “a lot of people are embarrassed” (Imogen) and “a lot of people put off going because they feel embarrassed” (Jane). Like pain, embarrassment was reported as a downside to screening that was weighed up against the core benefit of the test (lifesaving).

“It’s. . .a bit embarrassing, but do you know what? That could save your life” (Paula)

As well as general feelings of embarrassment, women felt that a factor that put them and/or others off screening was feelings of insecurity about their bodies. This included concerns about being “normal” (Jane) or “perfect” (Zara, Jane, Yvonne).

“I think most people everyone you see in a bikini, or whatever they just look perfect, and then I look at myself and go ‘Oh God! Should I look like that?’ You think ‘is it normal?’ ‘What is normal?” (Jane)

Some suggested that women from some cultural backgrounds might be reluctant to attend due to cultural norms around sex and nudity. Vera, who is Iranian, said that women she knows are “ashamed of their body,” and Chantelle, who is of South Asian heritage, said that at the age of 25 “most people have not had anyone look at that part of their body except for themselves.”

#### Barrier: Vulnerability/violation

Women reported that a contributing factor to experiences of cervical screening were feelings of being “vulnerable” (Renee, Wendy, Elspeth, Jane, Lauren), feeling “violated” (Nadia) and finding screening “traumatic” (Rebecca).

“It’s not really comfortable as you need to be like half naked, so no *one* wants that.” (Sharon)

Renee suggested that some women might not know that they can request a female practitioner and that this may be a barrier. Some suggested that past “sexual assault and sexual violence” (Victoria) may be a reason that some women do not attend, including one woman who finds the screening process brings up her own memories of sexual violence:

“I have had experiences with men in the past which haven’t been very nice. . .I think that certain things can be a bit triggery. . .I think that would be something that maybe is a big deal for a lot of people, and that is maybe why they don’t go.” (Jane)

##### Mitigating factor: Speed of procedure

Women said that the cervical screening procedure is over quickly, and that this made it easier to choose to attend. Women reported that “it’s just three minutes” (Paula), “five minutes” (Lucille, Beth, Sharon, Jane) “quick” (Orla, Queenie, Vera, Yvonne, Anita, Maureen, Lauren) and that it does not go on for “long” (Becky, Victoria). This was weighed up against both negative aspects of attending screening (e.g. pain, embarrassment) and negative consequences of not attending (e.g. undiagnosed cancer) when explaining attendance decision-making.

“I’d rather have a couple of minutes of embarrassment and discover I’ve got something wrong and fix it, rather than letting something fester and become ill.” (Olivia)

#### Mitigating factor: Person-centred care

Women referred to interactions with staff as relevant to their screening experiences. Participants expressed that having a supportive relationship with a clinician, in which they felt that they were interacted with in a respectful, humanising manner, was very important for their sense of safety and for reducing negative experiences associated with cervical screening. The way that staff related to women was understood to be able to make the difference between a positive and a negative screening experience, and thus to be able to mitigate against the potential downsides of screening. Some women described staff as “lovely” (Rosie, Renee), “wonderful” (Beth), “super nice” (Dana), “brilliant” (Elspeth) and “compassionate” (Renee).

Women felt positive about experiences in which staff spent time talking with them, and “explain everything as you go along” (Dana).

“I’m fortunate. I had a wonderful nurse practitioner who sat me down beforehand and had a conversation with me” (Beth)

This was contrasted with staff who “just get on with it” (Jane), and those who were described as “abrupt” (Jane), “impatient” (Olivia) or “forceful” (Cindy). One woman recounted an experience in which she was not listened to by a nurse and suggested that the outcome of this was that the procedure was more painful than it would otherwise have been.

“I once had a nurse and she was quite impatient and she was just wanting to get on with it very quickly. And I said I don’t think I’m relaxed enough yet and she just kind of went with the clamp and I wasn’t ready for it, it really hurt!” (Olivia)

This suggests that although women want the procedure to be over quickly, they benefit from having time during the appointment to share concerns and fears and be listened to. Women wanted to have their wishes, and their own understandings of their bodies and needs, taken into consideration by healthcare professionals. They wanted to experience themselves as being heard and their concerns taken seriously, rather than dismissed. Rebecca, who has had cervical cancer and is the only interviewee who does not currently attend as recommended, said one reason she has stopped attending was that she felt that the nurses who performed her screening had “complete indifference” to her needs.

“The absolute agony I went through, put me off going. . .because it was getting more traumatic to go to it each time, because you knew it was coming, and the ladies that I saw, had absolutely zero sympathy. . .It is just too traumatic from a psychological point of view.” (Rebecca)

#### Mitigating factor: Continuity of care

As well as the importance of being listened to during the screening encounter, some women suggested that being screened by people with whom they have an ongoing relationship, and in a place that is “familiar” (Orla) rather than “alien” (Elspeth), is helpful.

“I booked myself into my family doctor. I knew my nurses, so it was no problem at all.” (Anita)

“You know your GP, and you know the nurses at your GP’s. . . so it’s a bit more comfortable than just getting anybody from the hospital if that makes sense.” (Cindy)

#### Mitigating factor: Knowledge of screening experience

Women suggested that knowledge about “what they will experience” (Jean) would be helpful for decision-making about screening attendance. This included information about “the process” (Cindy), that “it lasts about this long” (Nadia), and that “you can see a female nurse or female doctor” (Renee). Renee added that reassurance “that it is not a hideous test” would be useful to encourage attendance. Participants suggested that it would “put people at ease” if the screening invitation letter were to include “what we are going to do to you” (Nadia) and “we are going to do this, this and this” (Cindy). Others mentioned that women do not know “what to expect” (Sharon, Olivia, Jean, Gillian), and that fear of “the unknown” (Sharon) was a potential barrier to attendance. Participants also perceived that improving openness and information about screening could reduce embarrassment by helping women to feel that screening is “normal” (Elspeth, Denise). However, some participants felt that detailed anatomical detail of the procedure can be off-putting. Renee suggested that, while women need to know what will happen to them when they attend screening, some leaflets are “a bit too invasive” and “could be written with a little more sensitivity.” Victoria said she chose not to read the booklet she received with her screening invitation letter because she did not want “the gory details.”

Overall, women who reported that the screening procedure itself is over quickly felt positive about this. However, women also felt that it was important that staff took enough time with them to explain what was happening and put them at ease, and that it was helpful to be screened in a familiar environment by somebody they had a pre-existing relationship with. Some felt that this made the physiological process itself easier because they were more relaxed. Women also suggested that appropriate knowledge about the screening process can be reassuring and encourage attendance, although some felt that too much detail about the procedure is unhelpful. This suggests that, like knowledge of the purpose of screening, knowledge of the process is mediated by individual beliefs and meaning-making.

## Discussion

This qualitative analysis is focussed on factors which influence women’s cervical screening decision-making. This study makes a novel contribution by empirically exploring the paradox between commonly reported barriers and screening behaviour. By focusing on how women manage and mitigate these perceptions, our findings extend beyond barrier identification to emphasising facilitators of engagement.

Our analysis identified two core themes: perceived purpose of screening and screening experiences. The importance of women’s perceptions of both the purpose and experience of cervical screening in their decision-making about screening attendance aligns with previous research (Armstrong et al., 2012; e.g. Chorley et al., 2017; Waller et al., 2009). However, the current study builds on this to offer novel understanding about how negative perceptions can be overcome. Our analysis indicates that women perceive that knowledge of the purpose of screening is important for informed decision-making. Speed of the procedure, person-centred care, continuity of care, and knowledge of the screening process, were all understood as mitigating against negative screening experiences.

Most participants believed that improved knowledge of cervical screening purpose would increase uptake. Women’s perceptions that knowledge is important for their screening decision-making should be understood within the context that women felt they and other women had a lack of relevant knowledge about both the purpose and process of screening. During the course of their interview, 93% (*n* = 41) women mentioned that they feel uninformed and/or that they feel that there is a lack of information about HPV, cervical cancer and cervical screening available to women. Research has consistently found poor knowledge of HPV and HPV screening (e.g. Kola-Palmer and Dhingra, 2020; Mulcahy Symmons et al., 2021; Patel et al., 2018), and poor knowledge of the link between HPV and cervical cancer (Kola-Palmer et al., 2022; Taratula-Lyons and Hill, 2024). In the present study, women gave lack of knowledge about cervical cancer and HPV as a reason for having avoided attending screening in the past and suggested that this is a reason that other women do not attend.

However, some explained that they perceived a sexual stigma associated with HPV and cervical cancer. This suggests that, for some women, knowledge of HPV as a sexually transmitted infection, and cervical screening as an HPV test, may not increase uptake. This finding aligns with research suggesting that knowledge that cervical screening is a test for HPV, and that HPV is a sexually-transmitted infection, may be a barrier to screening for some women (Patel et al., 2018). This suggests that knowledge of cervical cancer prevention strategies plays a dual role in either encouraging or discouraging screening uptake, depending on individual meaning-making, which may be culturally influenced and impacted by individual factors and degree of understanding. This novel insight extends existing research by highlighting the complex and individualised impact of understanding on screening behaviour. These findings extend those of Chorley et al. (2017), highlighting the importance of personal beliefs in screening decision-making.

For most participants, however, the only potential reasons for non-attendance related to the experience of screening, which included pain, embarrassment and a sense of vulnerability/violation. These negative experiences were understood to be potentially mitigated, or avoided altogether, when clinicians (a) performed the procedure fast, and (b) spent time interacting with the woman on a subject-subject basis, which included listening to and taking into account her own knowledge of her body and creating an environment in which women felt that they were engaged with as human beings, rather than only as bodies. Women value screening staff who personally engage and take the time to understand the individual perceptions and concerns of each woman. Our analysis shows that positive relational healthcare encounters can increase women’s sense of safety and minimise the negative physical and psychological experiences associated with cervical screening. Women also value having screening performed by a person that they are familiar with, in a setting in which they feel comfortable. These findings draw attention to the importance of person-centred care and continuity of care in shaping women’s screening experiences.

Trust is an important component of patient-provider relationships, and increased patient trust is associated with increased use of preventive services, including cancer screening (Gupta et al., 2014). The relational aspect of trust is particularly relevant to women’s experiences of cervical screening. Armstrong et al. (2012) argue that poor quality encounters with screening staff during cervical screening is a betrayal of trust. Poor quality encounters were those that women described as cold, impersonal or even degrading. Patients trust in healthcare providers is shaped by three key factors: effective communication, a sense of being cared for, and perceived competence (Greene and Ramos, 2021). Of relevance to our findings, is that caring – where healthcare providers engage with patients as individuals and value their concerns – was seen as contributing to a high degree of trust, while provider self-interest or a purely procedural approach undermined trust. Where women feel they are treated as just another body and perceive that screening staff approach their role as a service to be completed rather than a patient-centred interaction, this is dehumanising (Gillibrand et al., 2025) and there is a clear potential for undermining trust and increasing disengagement. Continuity of care is also associated with increased patient trust (Mainous et al., 2001; Perriman et al., 2018). More broadly, trust can be understood as a mechanism for managing uncertainty and a way of framing the unknown as non-threatening (Frederiksen, 2014). In the context of cervical screening, trust enables women to navigate the discomfort, embarrassment and potential stigma associated with the procedure.

### Study limitations

These findings are timely and important, as cervical screening rates in the UK are declining. The qualitative methods of this study enabled a nuanced and deep analysis of the factors that influence screening decision-making beyond barriers to screening. However, while we recruited women with a broad range of cervical screening histories, the sample was drawn from respondents to an earlier survey who consented to be contacted for follow-up interviews. As such, the experiences, beliefs and perceptions reported here may represent women who are particularly interested in cervical screening. Women with histories of cervical disease, and women who attend screening as recommended, are overrepresented. Nevertheless, it is important that women with a range of clinical experiences are included in research. By not restricting recruitment only to those without cervical disease, the data were enriched by the perspectives of women who are often absent in research, providing a more rounded and grounded understanding of screening decision-making. Further research could test our findings through quantitative study of a generalisable sample of women in the UK.

### Synthesis and summary of findings

This study has found that women’s perceptions of the purpose and experience of cervical screening were central to their attendance decisions. Screening was widely understood as potentially lifesaving, which acted as a facilitator. Women believed that a lack of sense of personal risk, stigmatisation of HPV and cervical cancer and a fear of a cancer diagnosis, were barriers to attendance. While some participants had previously been deterred by these barriers, none cited them as current reasons for non-attendance. They did, however, believe that they may be reasons for other women’s non-attendance. Knowledge about cervical cancer, HPV and the role of cervical screening in cancer prevention was generally seen as a mitigating factor against these barriers, though for some, understanding that cervical cancer is caused by a sexually transmitted disease could reinforce stigma.

For most women in this study, the most important barrier for attending cervical screening was the experience of screening itself, which was perceived and/or experienced as potentially painful, embarrassing and violating. The one participant who did not attend as recommended at the time of the interview cited negative experiences with screening appointments and the procedure. Mitigating factors against negative experiences included a fast physical procedure, performed in the context of a trusting relationship with a healthcare professional. The relational context in which screening was experienced was perceived as important for making the experience less painful, embarrassing, and violating. This was understood as important for enabling women to choose to attend screening.

Our findings show that the meaning that women make of their knowledge of cervical screening, cervical cancer and HPV influences decision-making, for example, whether they see themselves as at risk from cervical cancer/HPV, and whether they associate cervical cancer/HPV with stigma, and whether they feel frightened or reassured by detailed knowledge of screening procedure. Knowledge is not an objective entity, but is interpreted through personal values, beliefs and meaning. Thus, even when women have access to the same factual cervical cancer prevention information, idiosyncratic perceptions, emotions, experiences and social contexts shape how they internalise and act on that knowledge.

It is therefore too simplistic to assume a linear relationship between more knowledge equating to better screening uptake, as our analysis shows that knowledge can both encourage and deter screening, depending on individual understanding and interpretation. Our findings suggest that to improve screening uptake, public health interventions must move beyond simply increasing knowledge and instead address the ways in which women make sense of screening information through personal meaning-making processes. Enhancing understanding requires more than information provision – it necessitates meaningful engagement that aligns with women’s values and lived experiences. In the context of women’s suggestions that person-centred care and continuity of care is important, it may be that providing opportunities for women to learn about HPV and cervical screening in a context in which they can ask questions and share concerns is appropriate. This could take place at different times and settings, in order to be relevant for different groups of women. These could include, for example, educational sessions delivered as part of the HPV vaccination programme and offered with screening invitations, as well as community-based initiatives to address the specific needs and concerns of different groups of women.

Women report negative perceptions and experiences of the screening process, but importantly some women still balance pros/cons in favour of screening uptake. As screening is an intermittent regular occurrence, experiences and perceptions of screening matter. Pain, embarrassment, stigma, shame and the intrusive nature of screening act as barriers. Women perceive person-centred care and continuity of care as an important mitigator of these barriers, and many women manage to tip the balance in favour of attending screening. This suggests that barriers alone are not enough to deter screening, thus cervical screening non-attendance cannot be truly explained by focusing on barriers alone (Waller et al., 2009), and there is a need to identify facilitators. Our findings suggest that trust may be an important concept for understanding attendance decision-making. Clear, accessible and patient-centred communication, and caring for patients in a way that recognises them as a whole person, plays a key role in building this trust (Asan et al., 2021; Greene and Ramos, 2021). Future research could explore in more detail the kinds of interactional encounters that foster trust during cervical screening appointments and consider systems of organising cervical screening to improve continuity of care.
